# Supplementary material for: Engineering new limits to magnetostriction through metastability in iron-gallium alloys
Source: Nat Commun. 2021 May 12;12:2757. doi: 10.1038/s41467-021-22793-x (PMC8115637; doi:10.1038/s41467-021-22793-x)
Supplement: Supplementary file 1 — Supplementary Information [file 41467_2021_22793_MOESM1_ESM.pdf]

Extended Data

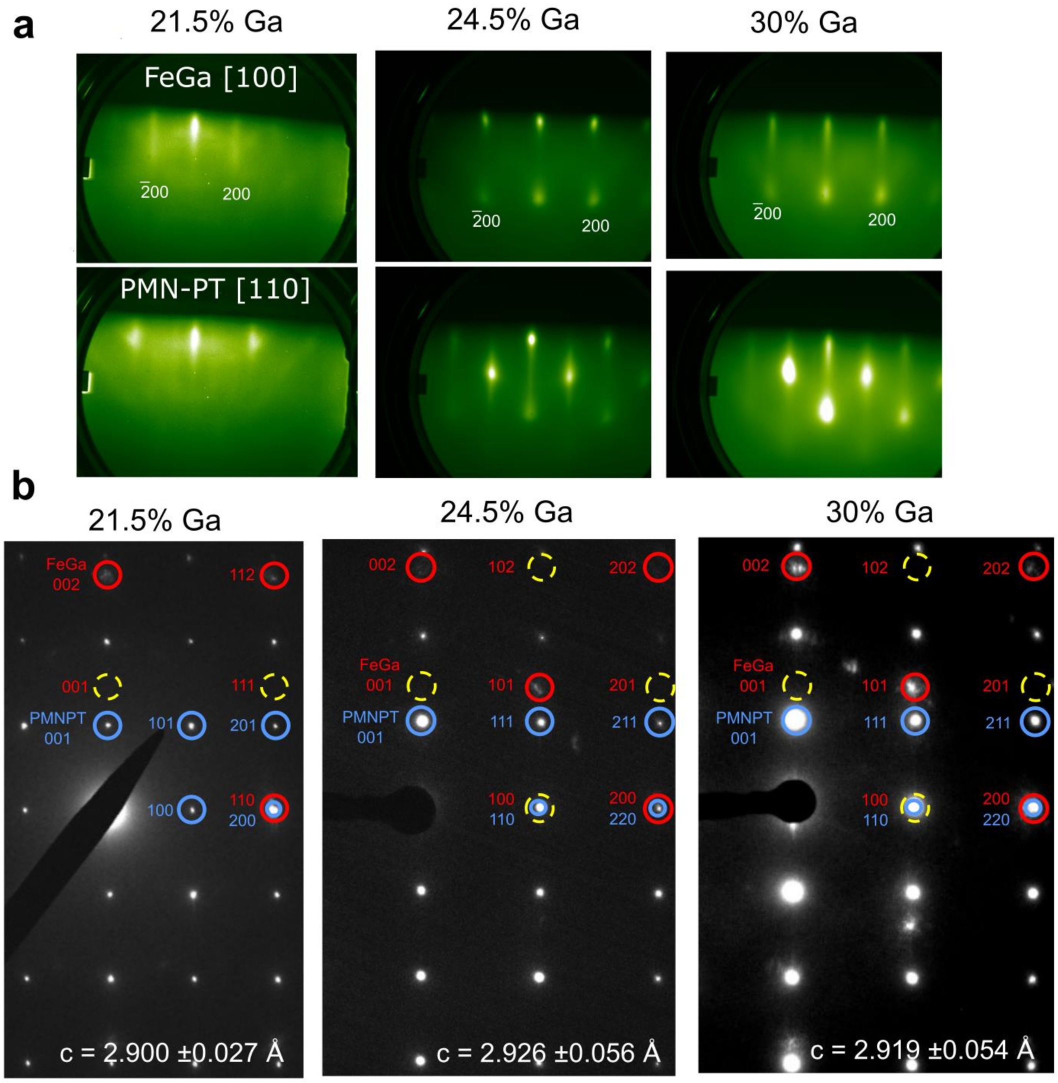

Supplementary Figure S1 | Diffraction data from $Fe_{1-x}Ga_x$. a, In-situ reflection high energy electron diffraction (RHEED) data on the $[110]_s$ azimuth of the PMN-PT substrate and the [100] of the $Fe_{1-x}Ga_x$ film. RHEED data shows no signal corresponding to the 100 peak of the $Fe_{1-x}Ga_x$, indicating that the films are in the disordered, A2-like phase. **b**, Select area electron diffraction (SAED) pattern of $Fe_{1-x}Ga_x$ thin films, which confirms $Fe_{1-x}Ga_x$ thin film is in the A2 phase rather than intermetallic B2 or $D0_3$ across different Ga concentrations. $Fe_{1-x}Ga_x$ Bragg peaks $Fe_{1-x}Ga_x$ (yellow dashed line) is extinct when sum of reciprocal lattice indices is odd, which occurs only in the A2 phase as detailed below in **Sup Note 1**. The out-of-plane lattice constants of the $Fe_{1-x}Ga_x$ film calculated from diffraction images are shown with error bars.

Supplementary Note 1 | Structure factor of $Fe_{1-x}Ga_x$ phases

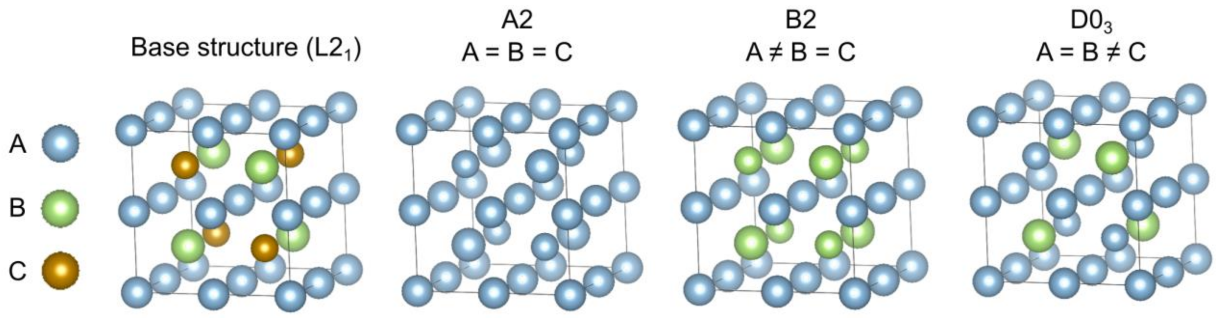

The A2, B2 and $D0_3$ structures can be represented in terms of the base structure ($L2_1$) with three atomic sites (A, B, C). For the solid solution A2, all three sites are equal. For B2, the B and C sites are equal, but distinct from the A site. Similarly, for $D0_3$, A and C are equal. The structure factor ($S(hkl)$) determines intensity of diffraction peaks at index (hkl). $S(hkl)$ for the base structure is:

$$S_{L2_1}(hkl) = \sum_i f_i \exp[-i\boldsymbol{b}_{hkl} \cdot \boldsymbol{r}_i]$$

$$= f_A\left[1 + e^{-i\pi h} + e^{-i\pi k} + e^{-i\pi l} + e^{-i\pi(h+k)} + e^{-i\pi(k+l)} + e^{-i\pi(h+l)} + e^{-i\pi(h+k+l)}\right]$$

$$+ f_B\left[e^{-\frac{i\pi}{2}(h+k+l)} + e^{-\frac{i\pi}{2}(3h+3k+l)} + e^{-\frac{i\pi}{2}(h+3k+3l)} + e^{-\frac{i\pi}{2}(3h+k+3l)}\right]$$

$$+ f_C\left[e^{-\frac{i\pi}{2}(h+k+3l)} + e^{-\frac{i\pi}{2}(3h+3k+3l)} + e^{-\frac{i\pi}{2}(h+3k+l)} + e^{-\frac{i\pi}{2}(3h+k+l)}\right],$$

where $\boldsymbol{b}_{hkl}, \boldsymbol{r}_i, f_i$ denote the reciprocal lattice vector at (hkl), basis vector and scattering factor respectively.

The [002] peak (equivalent to [001] peak for A2) is chemically sensitive to the A2 structure, as a peak can arise from either the B2 or $D0_3$ phases:

$$S_{L2_1}(002) = 4[2f_A - (f_B + f_C)]$$

$$S_{A2}(002) = 0$$

$$S_{B2}(002) = 8(f_A - f_B)$$

$$S_{D03}(002) = 4(f_A - f_B)$$

Similarly [113] peak is chemically sensitive to $D0_3$, as the peak can only arise from the $D0_3$ phase:

$$S_{L2_1}(113) = 4i(f_c - f_B)$$

$$S_{A2}(113) = 0$$

$$S_{B2}(113) = 0$$

$$S_{D03}(113) = 4i(f_A - f_B)$$

Therefore, the absence of a [002] for peak in SAED (**Fig. 1d**) is a good indicator of the pure A2 phase. Note that the [002] peak is equivalent to $[001]_{A2,B2}$, because A2 and B2 have a smaller conventional unit cell.

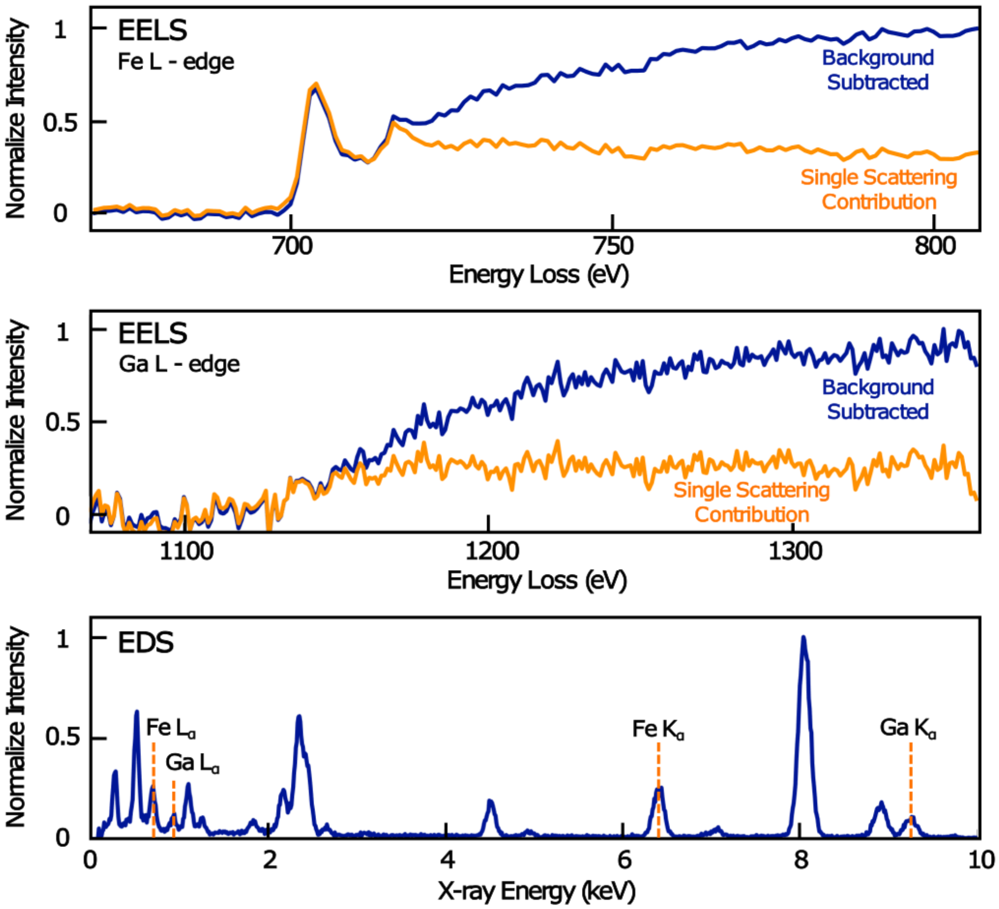

Supplementary Figure S2 | Spectroscopic Quantification of Gallium Concentration in $Fe_{1-x}Ga_x$ thin film. Background subtracted core-loss EELS spectra (blue) and single scattering contribution (SSC) spectra (yellow) of Fe L-edge (top) and Ga L-edge (middle)[1]. SSC spectra were obtained by Fourier-ratio deconvolution. In both cases deconvolution technique improves edge contour without any artifact or increase in noise level. (bottom) EDS spectrum acquired from $Fe_{1-x}Ga_x$ film. Fe and Ge K_α are well separated from other elements—apt for accurate quantification.

Supplementary Note 2 | Determination of Ga composition

The relative Ga/Fe concentration (x_{rel}) is equal to $x_{\mathrm{rel}} = \frac{I^1_{Ga}(\Delta)}{\sigma_{Ga}(\Delta,\beta)}\frac{\sigma_{\mathrm{Fe}}(\Delta,\beta)}{I^1_{Fe}(\Delta)}$, where I is integrated single scattering core-loss spectrum intensity, σ is inelastic cross-section, Δ is the integration window size (in eV), and β is EELS collection angle[1]. σ was calculated from Hartree-Slater model generalized oscillator strength[3]. This model showed a stable x_{rel} over ranges of Δ. With β = 34 mrad, and Δ_{Fe} = 30 eV, Δ_{Ga} = 50 eV, we report Ga concentration of 21.5 ± 3%. Note that the error is a lower bound calculated only from uncertainty of Hartree-Slater modeled σ.

For core-loss spectrum with energy loss well above low-loss region, the widening of spectrum due to plural scattering is well approximated by convolution of the single scattering contribution (SSC) and low-loss spectrum[1]. SSC was extracted by deconvolving of core-loss spectrum by low-loss spectrum and convolving by non-linear least square (NLLSQ) fitted zero-loss peak. The (de)convolution was handled by (dividing) multiplying in Fourier domain[2] (Fourier-ratio method). Background contribution was modelled by NLLSQ fitting linear combination of power laws to the signal preceding relevant edges[4]. The background was subtracted prior to extraction of SSC.

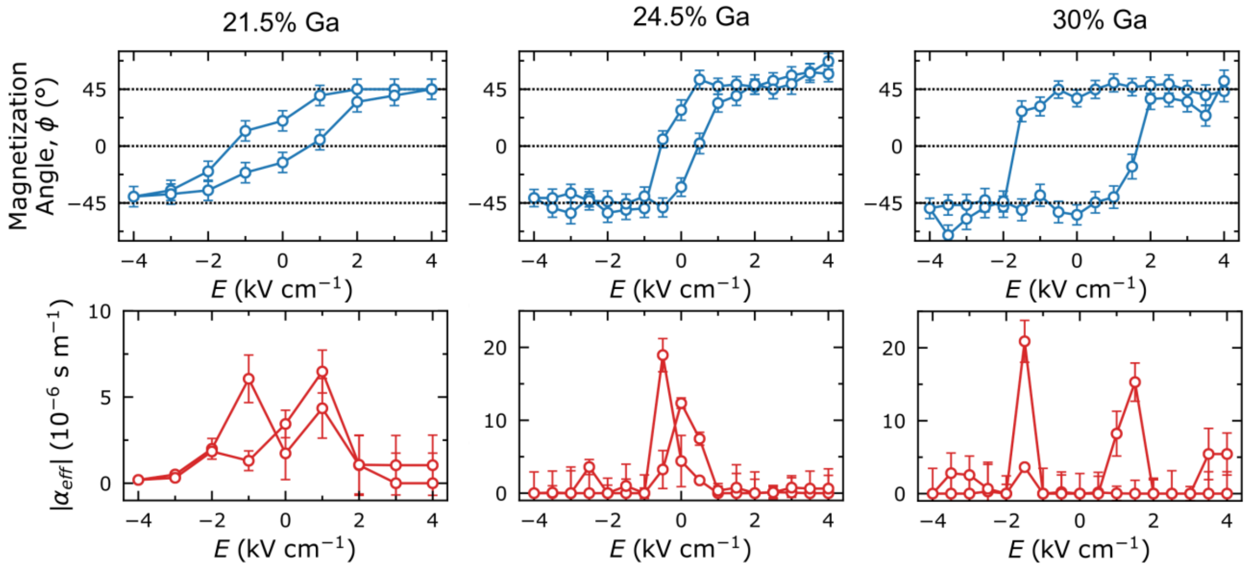

Supplementary Figure S3 | Complete magnetoelectric switching data. Example data taken at an electrical bias of up to ±400 V across the sample, used to reconstruct the hysteresis in magnetization direction and converse magnetoelectric coefficient. AMR curves taken as a function of field angle are fit to $\cos(2\theta)$ where the phase shift corresponds to the direction of magnetization, ϕ. The two saturated polarization states of the ferroelectric show a 90° phase shift in the curve, demonstrating a 90° switching of magnetization. The error bars represent the $\pm 5°$ angular resolution, the 1.0/0.5 kV cm^{-1} resolution in electric field, and one standard deviation of the fit to $\cos(2\theta)$. This measurement is done by poling the magnetization along the easy axis and using low-field directional AMR to probe the rotation of the uniaxial, strain-induced anisotropy. Thus, the apparent handedness is a result of the direction used in easy-axis poling and not indicative of the device itself, but is a direct probe of the anisotropy direction.

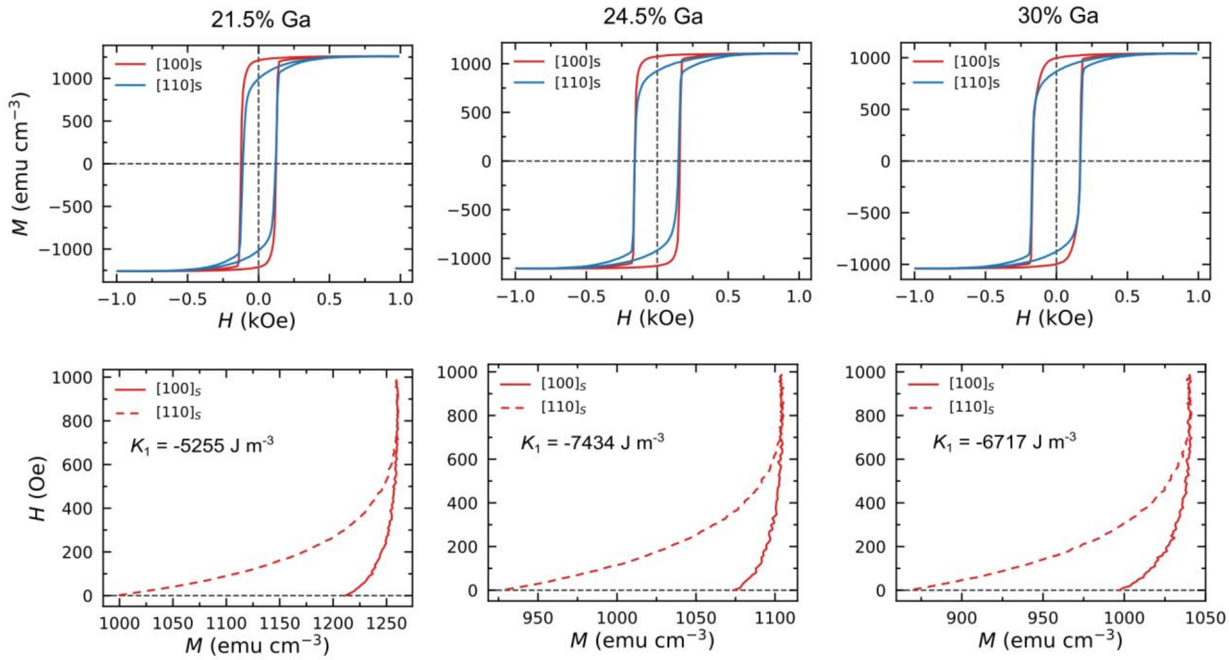

Supplementary Figure S4 | Calculation of anisotropies. Magnetic hysteresis loops of $Fe_{1-x}Ga_x$ samples and schematic of the integral, $A_{ijk} = \int_{M_r}^{M_S} H\,dM$, used to determine the magnetic anisotropy energy[2] through $A_{110} - A_{100} = K_1/4$. The values for anisotropy energy were used in the analytical solution as the required energy produced by strain at the switching voltage.

Supplementary Note 3 | Calculation of shear strain

Here, we calculate the distortion and corresponding strains that arise in pseudocubic (001) PMN-PT when the local polarization, **P**, switches by 109° from down (E = -4 kV cm^{-1}) to up (**Figure 3a**, E = +4 kV cm^{-1}). The distortion corresponding to the downward and upward polarization state is illustrated by the projection of PMN-PT unit cell onto the *x*-*y* plane of the substrate (**Figure 3b**). This distortion translates point r_1 at (x_1, y_1) to point r_2 at (x_2, y_2) and point r_3 at (x_3, y_3) to point r_4 at (x_4, y_4).

The unit cell parameters of PMN-PT[5] are $a = 4.017$Å and $\beta = 89.89°$, then the angle γ can be evaluated as $(90° - 89.89°)/2 = 0.055°$. Thus, points r_1 and r_3 are identified for the pseudocubic cell with polarization within the $(110)_{pc}$ plane: $x_1 = y_3 = -a\sin\gamma \approx -0.003856$ Å, $y_1 = x_3 = a\cos\gamma \approx 4.016998$Å. Similarly, points r_2 and r_4 are identified for the pseudocubic cell with polarization within the $(\bar{1}10)_{pc}$ plane: $x_2 = y_4 = a\sin\gamma \approx 0.003856$ Å, $y_2 = x_4 = a\cos\gamma \approx 4.016998$Å. When the polarization switches by 109° from down to up, the normal

strain changes as $\Delta\varepsilon_{xx}^{s} = \frac{x_4 - x_3}{x_3} = 0$, $\Delta\varepsilon_{yy}^{s} = \frac{y_2 - y_1}{y_1} = 0$ and the shear strain changes as $\Delta\varepsilon_{xy}^{s} = \frac{1}{2}\left(\frac{x_2 - x_1}{y_1} + \frac{y_4 - y_3}{x_3}\right) \approx 0.192\%$. Using an undistorted $(001)_{pc}$ cubic lattice as the reference, the shear strain of the x-y plane ε_{xy}^{s} associated with the downward and upward polarization should have the same magnitude but opposite signs. In this regard, ε_{xy}^{s} = -0.096% at *E* = -4 kV cm^{-1} and ε_{xy}^{s} = 0.096% at *E* = +4 kV cm^{-1}.

In our calculations, we assume these shear strains generated by the PMN-PT are completely transmitted to the $Fe_{1-x}Ga_x$ film across the heterointerface without loss. Due to the 45° misalignment between the in-plane lattices of these two materials (Figure 1c), the shear strains in the $(001)_{pc}$ plane of the PMN-PT are equivalent to a biaxial in-plane anisotropic strain in the (001) plane of the $Fe_{1-x}Ga_x$, $(\varepsilon_{11}^{\mathrm{FeGa}}, \varepsilon_{22}^{\mathrm{FeGa}})$ = (-0.096%, 0.096%) at *E* = +4 kV cm^{-1} and $(\varepsilon_{11}^{\mathrm{FeGa}}, \varepsilon_{22}^{\mathrm{FeGa}})$ = (0.096%, -0.096%) at *E* = -4 kV cm^{-1}. Furthermore, since ~20% of the ferroelectric domains in the PMN-PT undergo 109° switching when being poled from downward to upward[6], the average strain in the $Fe_{1-x}Ga_x$ can be estimated by multiplying the local strains above by the switching fraction of 20%: $(\varepsilon_{11}, \varepsilon_{22})$ = (-0.0192%, 0.0192%) at *E* = +4 kV cm^{-1} and $(\varepsilon_{11}, \varepsilon_{22})$ = (0.0192%, -0.0192%) at *E* = -4 kV cm^{-1}. This gives rise to an in-plane strain anisotropy $\varepsilon_{22} - \varepsilon_{11}$ = 0.038% at *E* = +4 kV cm^{-1}, which tends to align the magnetization along the [010]/[0$\bar{1}$0] axis of the $Fe_{1-x}Ga_x$ (ϕ = +45°), and conversely $\varepsilon_{22} - \varepsilon_{11}$ = -0.038% at *E* = -4kV cm^{-1}, which tends to align the magnetization along the [100]/[$\bar{1}$00] axis (ϕ = -45°). This agrees with the experimental observation, where ϕ ~ 50° at *E* = +4 kV cm^{-1} and ϕ ~ -40° at *E* = -4 kV cm^{-1}, with the $\pm$5° difference due to the misalignment of the device with the $[100]_s$ crystallographic direction.

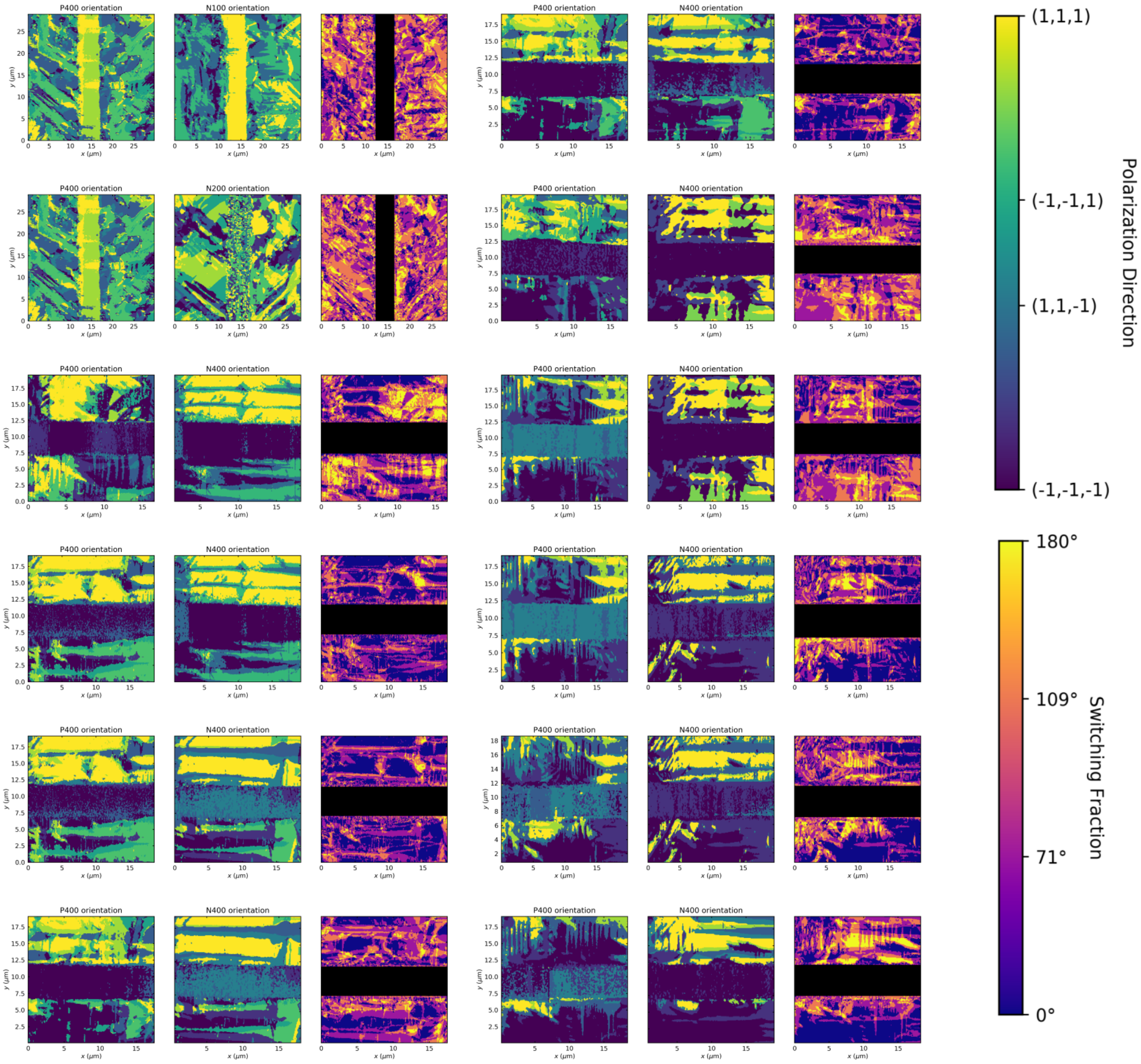

Supplementary Figure S5 | Domain fraction of ferroelectric switching. PFM switching map that allows us to experimentally determine $\eta_{109°}$. This map is made by overlaying PFM micrographs before switching (+4 kV cm^{-1}) and after switching -4 kV cm^{-1}) and calculating the 3D switching angle per pixel. The directions of the ferroelectric vectors were determined by combining in-plane and out-of-plane piezoresponse patterns before and after rotating the sample by 90° to allow for the determination of in-plane directionality.

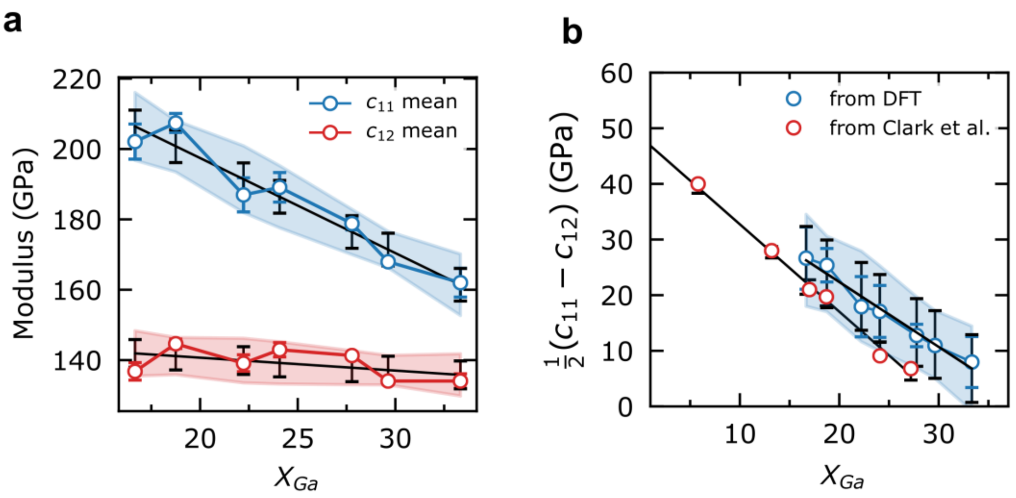

Supplementary Figure S6 | Density functional theory simulation of shear modulus. a, Simulated stiffness coefficients c_{11} and c_{12} from density functional theory of solid solution $Fe_{1-x}Ga_x$ alloys. The blue error bars correspond to the error of the calculation, the black error bars are one standard error of the linear fit, and the shaded area is the sum of the errors fixed about the trendline. **b,** Comparison of the calculated shear modulus values to bulk data extracted from ref [7]. The error bars correspond to the sum of the same quantities in part **a**, propagated through to $(c_{11} - c_{12})/2$. Our device is operated at room temperature, so the addition of temperature to the DFT results is simulated using a linear regression of compositionally dependent c_{11},c_{12} data from ref. [8]. Because 1) the trend of the bulk values are replicated well, 2) the bulk data is within the error bars of the DFT calculation, and 3) experimental values should be more precise than DFT results, the use of the experimental bulk mechanical values is justified for the calculation even though the bulk material is in a different phase.

Supplementary Note 4 | Detailed uncertainty in λ_{100}

The primary sources of error in the calculation of λ_{100} are 1) the magnetocrystalline anisotropy, 2) the values for strain, and 3) the stiffness tensor components c_{11} and c_{12}. For the anisotropy constant K_1, a 5% relative error is appropriate from tool calibration/misalignment. Regarding the switching fraction, $\sigma_\eta = 4\%$ from the measurements shown in Sup. Figure S5.

We use the bulk values of c_{11} and c_{12} because we believe that they present a more accurate estimate of the real stiffness coefficients than can be obtained from DFT calculations. While the DFT reproduces the trend and approximate order of magnitude well, the values themselves may

not be incredibly accurate, motivating us to pull more precise values from literature. As no uncertainty values are reported in ref. [7], where we obtain the c_{11}, c_{12} values, we use the standard error of the linear trendline in **Sup. Figure S6b** as the error for $\frac{(c_{11}-c_{22})}{2}$. The uncertainty in λ_{100} can then be defined as

$$\sigma_\lambda^2 = \left[\frac{1}{3}\frac{K_1}{\epsilon}\ \left(\frac{1}{c^2}\right)\right]^2 \sigma_c^2 + \left[\frac{1}{3c\epsilon}\right]^2 \sigma_{K_1}^2 + \left[\frac{1}{3}\frac{K_1}{c}\left(\frac{1}{\epsilon^2}\right)\right]^2 \sigma_\epsilon^2,$$

where $\epsilon = \eta\epsilon_{switch}$, and $c = \frac{c_{11}-c_{12}}{2}$.

Supplementary Note 5 | Micromagnetic Simulations

Micromagnetic simulations were performed in order to predict the dynamics of strain-enabled magnetization switching (i.e., $\partial\mathbf{m}/\partial t$) in a 15-nm-thick (001) $Fe_{1-x}Ga_x$ nanodisk grown on a PMN-PT(001) single crystal substrate. The simulations for Ga component x = 0.215, 0.245 and 0.3 are performed separately using MuMax[3] (version: 3.10β). This allows us to evaluate the energy dissipation arising from the interaction of a precessing magnetization with its surrounding lattice, $E_{\mathrm{d,m}}$. In single-domain nanomagnet, [11] $E_{\mathrm{d,m}} \cong V_{\mathrm{m}} \frac{\alpha\mu_0 M_{\mathrm{s}}}{\gamma} \int_0^{t_{\mathrm{eq}}} \left(\frac{d\langle\mathbf{m}\rangle}{dt}\right)^2 dt$. Here, $\langle\mathbf{m}\rangle$ is the volumetric average of the magnetization (**m**), V_{m} is the volume of the $Fe_{1-x}Ga_x$ nanodisk, α is the Gilbert damping coefficient, γ is the gyromagnetic ratio, and t_{eq} is the time required for the **m** to reach its equilibrium state. In our simulations, the equilibrium state is considered to be reached when $|\Delta\langle m_x\rangle/\Delta t^*|<10^{-4}$, where $\Delta\langle m_x\rangle$ denotes the change in $\langle m_x\rangle$ per reduced time step Δt^*. The diameter of the Fe*1-x*Ga*x* disk is set as 45 nm, the smallest projected size considering thermal stability at the experimentally observed values of magnetic anisotropy.

We assume that the 45 nm diameter $Fe_{1-x}Ga_x$ disk is overlain on one single ferroelectric domain of the PMN-PT substrate (which has a lower bound size[12] of ~300 nm) and that the ferroelectric domain undergoes 109° switching as E changes from +4 to -4 kV cm^{-1}. Such 109° domain switching is accompanied by a shear strain of 0.192%, which in effect applies a biaxial in-plane anisotropic normal strain $(\Delta\varepsilon_{11}, \Delta\varepsilon_{22})$ = (0.192%, -0.192%) (subscripts refer to the crystallographic frame of the $Fe_{1-x}Ga_x$) onto the $Fe_{1-x}Ga_x$ disk across the interface. As a result, the average magnetization of the $Fe_{1-x}Ga_x$ disk, which starts aligned along the [010] direction due to a pre-existing strain $(\varepsilon_{11}, \varepsilon_{22})$ = (-0.096%, 0.096%) from initial 0→+4 kV cm^{-1} poling, rotates to the new equilibrium direction [100] (or its energetically equivalent [$\bar{1}$00]) within t_{eq} ~ 4.0 ns for

$Fe_{0.785}Ga_{0.215}$, 4.44 ns for $Fe_{0.755}Ga_{0.245}$, and 4.69 ns for $Fe_{0.7}Ga_{0.3}$. Using these t_{eq} and the simulated $\partial \mathbf{m}/\partial t$ (see Fig. S7 below), the calculated $E_{\mathrm{d,m}}$ are ~0.6 aJ, ~0.9 aJ, and ~0.8 aJ, respectively.

The following materials parameters were used: for $Fe_{0.785}Ga_{0.215}$, K_1 = -5.255 kJ m^{-2}, M_s = 1.26×10^{6} A m^{-1}, B_1 = -1.368×10^{7} J m^{-3}; for $Fe_{0.755}Ga_{0.245}$, K_1 = -7.434 kJ m^{-2}, M_s = 1.104×10^{6} A m^{-1}, B_1 = -1.936×10^{7} J m^{-3}; for $Fe_{0.7}Ga_{0.3}$, K_1 = -6.717 kJ m^{-2}, M_s = 1.04×10^{6} A m^{-1}, B_1 = -1.75×10^{7} J m^{-3}, where K_1 and M_s are experimental values (Fig. S4). For all three cases, exchange coefficient A_{ex}= 1.8×10^{-11} J m^{-1}, α = 0.017,[13] γ = 2.25×10^{5} Hz (A m^{-1})$^{-1}$,[14] A cell size (Δx, Δy, Δz)= (1 nm, 1 nm, 2.5 nm) is used in all simulations. A reduced time step Δt^*(~0.0057 for $Fe_{0.785}Ga_{0.215}$, ~0.005 for $Fe_{0.755}Ga_{0.245}$, ~0.0047 for $Fe_{0.7}Ga_{0.3}$, corresponding to 20 fs in real unit) is used. Our testing showed that using half of the Δt^* would yield the same results.

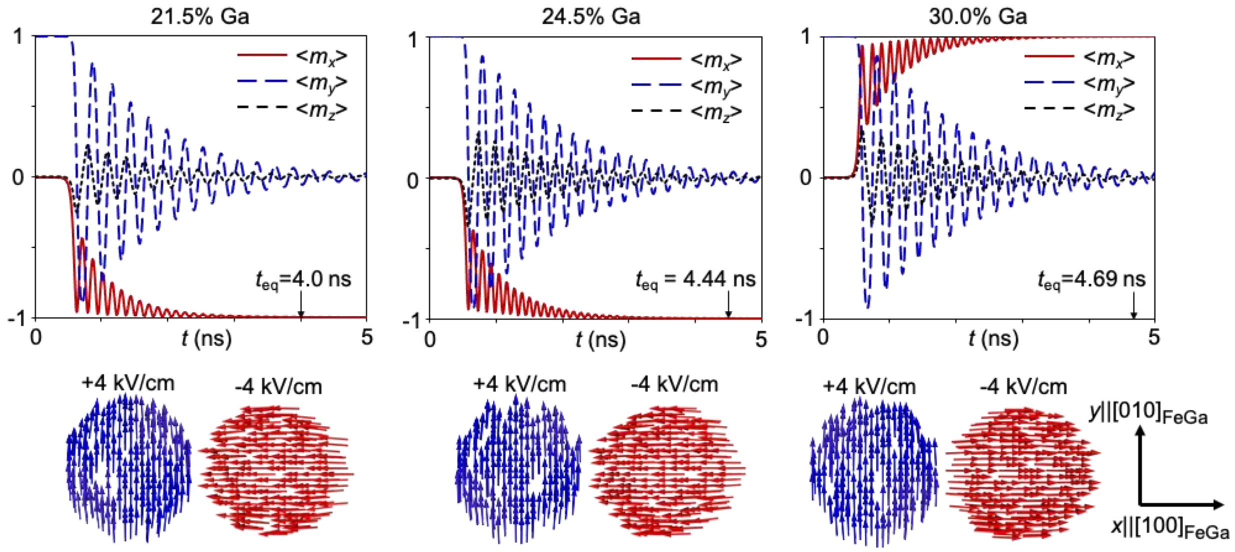

Supplementary Figure S7 | Electric-controlled magnetization switching in a single-domain Fe1-xGax nanodisk by phase-field simulations. Temporal evolution of <*m*$_i$> (*i* = *x*,*y*,*z*) in the $Fe_{0.785}Ga_{0.215}$, $Fe_{0.755}Ga_{0.245}$, and $Fe_{0.7}Ga_{0.3}$ disk (diameter: 45 nm; thickness: 15nm) upon a +4→-4 kV cm^{-1} poling to the PMN-PT substrate underneath. Local magnetization distributions of the $Fe_{1-x}Ga_x$ at the initial state (*t* = 0) and the equilibrium state (*t* = t_{eq}) are placed below correspondingly. The applied electric field is +4 kV cm^{-1} at *t* = 0, and then switched to -4 kV/cm at *t* > 0. The (001) $Fe_{1-x}Ga_x$ disk is on top of one single ferroelectric domain of the (001) PMN-PT substrate. According to the analysis in Sup. Note 3, the $Fe_{1-x}Ga_x$ disk is subject to a biaxial in-

plane anisotropic strain of (ε_{xx}, ε_{yy}) = (-0.096%, 0.096%) at the initial state that arises from a 0→+4 kV cm^{-1} poling. The 109° ferroelectric domain switching during the +4→-4 kV cm^{-1} poling changes the strain state in the $Fe_{1-x}Ga_x$ disk to (ε_{xx}, ε_{yy}) = (0.096%, -0.096%), which leads to a nominal 90° magnetization switching. The strain on the (001) PMN-PT surface is assumed to rise instantaneously after electric field was switched to -4 kV cm^{-1} for simplicity.

Supplementary References:

1. Egerton, R. F. *Electron Energy-Loss Spectroscopy in the Electron Microscope*. (Springer US, 2011).
2. Rafique, S., Cullen, J. R., Wuttig, M. & Cui, J. Magnetic anisotropy of Fe1-xGax alloys. *J. Appl. Phys.* **95**, 6939–6941 (2004).
3. Rez, P. Cross-sections for energy loss spectrometry. *Ultramicroscopy* **9**, 283–287 (1982).
4. Cueva, P., Hovden, R., Mundy, J. A., Xin, H. L. & Muller, D. A. Data Processing for Atomic Resolution Electron Energy Loss Spectroscopy. *Microsc. Microanal.* **18**, 667–675 (2012).
5. Noheda, B., Cox, D. E., Shirane, G., Gao, J. & Ye, Z.-G. Phase diagram of the ferroelectric relaxor (1-x)$PbMg_{1/3}Nb_{2/3}O_3$-x$PbTiO_3$. *Phys. Rev. B* **66**, 054104 (2002).
6. Yang, L. *et al.* Bipolar loop-like non-volatile strain in the (001)-oriented $Pb(Mg_{1/3}Nb_{2/3})O_3$-$PbTiO_3$ single crystals. *Sci. Rep.* **4**, 4591 (2014).
7. Clark, A. E. *et al.* Extraordinary magnetoelasticity and lattice softening in bcc Fe-Ga alloys. *J. Appl. Phys.* **93**, 8621–8623 (2003).
8. Petculescu, G., Hathaway, K. B., Lograsso, T. A., Wun-Fogle, M. & Clark, A. E. Magnetic field dependence of galfenol elastic properties. *J. Appl. Phys.* **97**, 10M315 (2005).
9. Guo, X. *et al.* Electrical field control of non-volatile 90° magnetization switching in epitaxial FeSi films on (001) 0.7[$Pb(Mg_{1/3}Nb_{2/3})O_3$]-0.3[$PbTiO_3$]. *Appl. Phys. Lett.* **108**, 042403 (2016).

10. Zhang, S. *et al.* Electric-Field Control of Nonvolatile Magnetization in $Co_{40}Fe_{40}B_{20}/Pb(Mg_{1/3}Nb_{2/3})_{0.7}Ti_{0.3}O_3$ Structure at Room Temperature. *Phys. Rev. Lett.* **108**, 137203 (2012).

11. Peng, R.-C., Hu, J.-M., Chen, L.-Q. & Nan, C.-W. On the speed of piezostrain-mediated voltage-driven perpendicular magnetization reversal: a computational elastodynamics-micromagnetic phase-field study. *NPG Asia Mater.* **9**, e404 (2017).

12. Viehland, D. D. & Salje, E. K. H. Domain boundary-dominated systems: adaptive structures and functional twin boundaries. *Adv. Phys.* **63**, 267–326 (2014).

13. Azovtsev, A. V. & Pertsev, N. A. Coupled magnetic and elastic dynamics generated by a shear wave propagating in ferromagnetic heterostructure. *Appl. Phys. Lett.* **111**, 222403 (2017).

14. Parkes, D. E. *et al.* Magnetostrictive thin films for microwave spintronics. *Sci. Rep.* **3**, 2220 (2013).
